# Burnout among Syrian medical residents: A cross-sectional study using the burnout assessment tool (BAT)

**DOI:** 10.1371/journal.pone.0350426

**Published:** 2026-06-15

**Authors:** Mohammad Al-Jawad, Zain Douba, Somayya Tabasho, George Zakhour, Nawal Tijan, Nour Srewell, Lubna Haj Bakour, Layan Sabbagh, Hasan Ashkar, Shahd Almansour, Malak A.L. Saleh, Abdalrazzak Helaal, Waseem Zakaria

**Affiliations:** 1 Faculty of Medicine, University of Aleppo, Aleppo, Syria; 2 Department of Hematology, Syrian Ministry of Health, Aleppo, Syria; 3 Faculty of Medicine, Latakia University, Latakia, Syria; 4 Faculty of medicine, Homs university, Homs, Syria; 5 Faculty of Medicine, Hama university, Hama, Syria; 6 Department of Gastroenterology Central Idleb Hospital, Faculty of Medicine, Free Aleppo University, Aleppo, Syria; 7 Department of Internal medicine, Syrian Board of Medical Specialties, Idlib, Syria; College of Nursing, Qassim University, SAUDI ARABIA

## Abstract

**Introduction:**

Burnout syndrome, described by Herbert Freudenberger in 1974, is a psychological condition caused by chronic workplace stress, particularly in healthcare. It is characterized by emotional exhaustion, depersonalization, and a reduced sense of achievement. Burnout has become prevalent among healthcare workers, especially physicians and residents, with rates often exceeding 50%. In Syria, ongoing conflict has further stressed the healthcare system, exacerbating burnout among medical residents. This study aims to assess the prevalence and contributing factors of burnout among Syrian medical residents using the Burnout Assessment Tool (BAT), a more precise evaluation tool than traditional measures.

**Methods:**

A cross-sectional study was conducted from April 18 to May 1, 2025, among resident physicians in hospitals across six Syrian governorates. Licensed physicians with at least one year of clinical experience were eligible. Data were collected via an online questionnaire (KoboToolbox) assessing demographics, work conditions, burnout symptoms (BAT-23), job satisfaction, coping strategies, and workplace challenges. The minimum required sample size was 373. SPSS v25 was used for analysis. Non-parametric tests were applied due to non-normal data distribution.

**Results:**

A total of 550 medical residents participated, with the majority being female and in their early residency years. Participants primarily worked in governmental hospitals. Using the BAT23, 50% of participants were in the high-risk (Red) zone, with exhaustion being the most prevalent (76.7%). Cognitive impairment and emotional impairment were also common. Specialties like internal medicine and surgery showed higher burnout rates, with factors like workload, training, and work environment strongly linked to burnout. No significant differences were found based on Sex, age, or marital status.

**Discussion:**

The BAT provided a comprehensive assessment of burnout, highlighting exhaustion as the main driver, while mental health was less impacted. Mid-level residents (years 2–4) faced the highest burnout rates due to increased responsibilities without senior mentorship. High-stress specialties like internal medicine, surgery, pulmonology, and anesthesia were more vulnerable to burnout. The study underscores the need for systemic reforms, including improved workload distribution, better training, and mental health support.

**Conclusion:**

The study reveals high levels of burnout among Syrian medical residents, particularly in exhaustion and cognitive impairment. The highest rates were seen in mid-level residents and high-stress specialties. Structural reforms, such as better work conditions, training, and mental health support, are essential for mitigating burnout and ensuring the sustainability of Syria’s healthcare system.

## Introduction

Burnout syndrome was first described in 1974 by Herbert Freudenberger as a psychological condition that gradually develops due to prolonged exposure to chronic workplace stressors, especially those related to interpersonal interactions [1]. It was officially recognized by the World Health Organization in 2019 as an occupational phenomenon in the International Classification of Diseases (ICD-11), characterized by chronic workplace stress that has not been successfully managed. [2] Burnout has become highly prevalent among both physicians and residents, with reported rates often reaching or exceeding 50% [3].

Emotional exhaustion and irritability at work can contribute to various mental health issues, notably burnout, which includes key components such as emotional exhaustion, depersonalization, and reduced sense of personal achievement [4]. Four key contributors to burnout among primary health care workers have been identified: [1] limited autonomy in managing work conditions and making decisions; [3] time constraints that make physicians feel valued mainly for their productivity; [4] disorganized and inefficient workplaces where physicians are burdened with administrative or routine tasks; and misalignment between physicians and leadership in terms of values, goals, mission, and financial recognition [5].

Burnout has been linked to various physical health issues such as cardiovascular diseases, type 2 diabetes, musculoskeletal pain, chronic fatigue, and gastrointestinal and respiratory problems. Psychologically, it is associated with insomnia, depression, use of psychiatric medications, and even suicidal ideation. These serious outcomes highlight burnout as a critical public health concern that requires urgent preventive efforts in the workplace [6].

In Syria, years of conflict have repeatedly targeted the health sector: by March 2025 only 57% of hospitals and 37% of primary health centers were fully functional, 246 facilities faced imminent closure, and up to 70% of the workforce had fled abroad, forcing the remaining clinicians to cover extensive service gaps with constrained resources [7]. Intensified violence since November 2024 displaced over 882,000 Syrians and coincided with 37 documented attacks on health premises, amplifying the trauma load on the residents who remain on the front lines [8]. More than 16.7 million people now depend on humanitarian health support, meaning residents routinely provide care in overstretched, emergency-driven environments where burnout carries immediate population-level consequences [9].

On the other hand, in Syria, the prolonged crisis and ongoing conflict have had a profound impact on all aspects of life, including healthcare and education, leading to a general decline in living conditions. These challenges have placed additional pressure on medical residents and physicians, who are not only endured the effects of war but also had to compensate for the collapsing healthcare system by taking on extra responsibilities as care providers [3]. While burnout is a global concern among healthcare professionals, its impact in conflict-affected settings like Syria is especially critical. Therefore, considering these challenges, it has become essential to assess burnout syndrome among medical residents in Syria.

To achieve this, we employed the Burnout Assessment Tool (BAT), a recently developed and validated instrument that offers a more precise evaluation of burnout compared to traditional measures. The BAT allows for assessing the syndrome, along with its core dimensions and secondary symptoms [10]. By adopting this updated conceptualization of burnout, the purposes of this study were to investigate the prevalence of burnout syndrome among resident physicians of all specialties in Syria and to identify the factors influencing its occurrence among resident physicians.

## Methods

### Study design and setting

This cross-sectional study was conducted between April 18 and May 1, 2025, targeting resident physicians across hospitals in six Syrian governorates: Aleppo, Damascus, Homs, Hama, Latakia, and Idlib.

### Participants

Eligible participants were licensed medical doctors practicing in Syria with at least one year of clinical experience and currently enrolled in a postgraduate residency training program (years 1–6). Doctors who were on recent leave or had less than one year of experience were excluded.

### Sampling frame and recruitment

The sampling frame consisted of all resident physicians officially enrolled in postgraduate training programs across the six targeted governorates. A master list of eligible residents was obtained from residency program coordinators and training directors at the Ministry of Health (MOH) and Ministry of Higher Education (MOHE) hospitals in each governorate. This list included 620 actively enrolled residents.

Eligible residents were invited to participate via an online survey hosted on KoboToolbox. Invitations were distributed using a multi-channel approach: (1) program coordinators and training directors forwarded the survey link through official institutional email lists; (2) follow-up reminders were sent via WhatsApp groups maintained by residency programs; and (3) direct messages were sent to individual residents who had not responded after two weeks. The survey link was active for 14 days (April 18 – May 1, 2025), with reminder messages sent at day 3, day 7, and day 10 to non-responders.

Of the 620 eligible residents contacted, 550 completed the survey in full, yielding a response rate of 88.7%. All responses were anonymous, and no personal identifiers were collected. The high response rate was likely facilitated by the involvement of program coordinators who encouraged participation and by the use of familiar communication channels (WhatsApp) commonly used for residency coordination in Syria.

### Sample size calculation

The minimum required sample size was calculated using Calculator.net with standard assumptions (95% confidence level, 5% margin of error, estimated total population of medical residents in Syria (12,000), and 50% expected proportion. This yielded a minimum target of 239 participants. The sampling frame for this study consisted of 620 eligible residents across six governorates, and the final sample of 550 exceeded the calculated target.

### Data collection instrument

Data were collected using an online questionnaire via KoboToolbox, including demographic information, work conditions, and burnout symptoms assessed using the Burnout Assessment Tool (BAT-23) on a 5-point Likert scale. Additional questions explored job satisfaction, coping strategies, and workplace challenges.

### Human ethics & consent to participate

This study was approved by the Institutional Review Board (IRB) of Faculty of Medicine, University of Aleppo (Approval No: 2527) and complied with the Declaration of Helsinki. Given the fully anonymized nature of the collected data (no personal identifiers), the ethics committee waived the requirement for individual written consent. Completion and submission of the online survey served as implied consent.

### Statistical analysis

Statistical analysis was performed using SPSS version 25. Since the continuous variables were not normally distributed (as confirmed by Shapiro-Wilk and Kolmogorov-Smirnov tests), non-parametric tests were used: Kruskal-Wallis for comparing groups, Spearman’s rank correlation for ordinal data, and Chi-square, Fisher’s exact, or Monte Carlo simulations (99% confidence, 10,000 iterations) for categorical variables. A p-value of <0.05 was considered statistically significant.

## Results

A total of 550 medical residents participated in this study. The majority were female (56.9%) and single (63.1%). Most participants specialized in internal medicine (52.9%), followed by surgical (30.2%) and clinical specialties (6.9%). The most common specialties included General Internal Medicine and Pediatrics (15.3% each), and Gynecology and Obstetrics (11.8%).

Participants were mainly from Aleppo (32.0%), Idlib (26.0%), and Damascus (14.0%). Nearly all participants worked in governmental hospitals (97.5%), with 56.9% affiliated with the Ministry of Higher Education (MOHE) and 43.1% with the Ministry of Health (MOH).

In terms of training, the largest proportion of participants were in their 2nd year of residency (36.5%), followed by those in their 3rd year (27.1%). The median age across the cohort was 27 years (interquartile range [IQR] = 2), and the typical number of night shifts per month was 9 (IQR = 4), suggesting a moderate to high workload Table 1.

**Table 1:** Baseline characteristics.

Variable	Percent	Frequency
Sex		
Male	43.1%	237
Female	56.9%	313
Marital status		
Single	63.1%	347
Married	16.7%	92
Married with kids	19.5%	107
Divorced	0.7%	4
Specialty		
*Internals*		
Cardiology	4.0%	22
Endocrinology	2.9%	16
Family Medicine	2.4%	13
Gastroenterology	3.6%	20
General Internal Medicine	15.3%	84
Hematology	0.5%	3
Nephrology	1.6%	9
Neurology	2.0%	11
Pediatrics	15.3%	84
Pulmonology	3.5%	19
Rheumatology	1.1%	6
*Surgeries*		
Cardiac Surgery	0.9%	5
General Surgery	5.3%	29
Gynecology and Obstetrics	11.8%	65
Neurosurgery	1.5%	8
Ophthalmology	3.6%	20
Orthopedic Surgery	5.1%	28
Pediatric Surgery	2.0%	11
Plastic Surgery	0.2%	1
Thoracic Surgery	0.2%	1
Urology	2.0%	11
Vascular Surgery	1.3%	7
*Clinics*		
Dermatology	0.7%	4
ENT	2.5%	14
Psychiatry	1.3%	7
*Other*		
Anesthesia and Resuscitation	2.2%	12
Laboratory Medicine	0.2%	1
Oncology	0.7%	4
Pathology	2.4%	13
Radiology and Medical Imaging	1.8%	10
Other	2.2%	12
Province		
Aleppo	32.0%	176
Damascus	14.0%	77
Homs	11.1%	61
Idlib	26.0%	143
Latakia	10.2%	56
Other governorates	6.7%	37
Type of recruitment		
Governmental hospital	97.5%	536
Private hospital	2.5%	14
Type of hospital		
MOHE	56.9%	313
MOH	43.1%	237
Year of residency		
1	6.4%	35
2	36.5%	201
3	27.1%	149
4	23.5%	129
5	6.0%	33
	**Median**	**IQR**
Age	27	2
Number of night shifts per month	9	4

Note: “Other governorates” includes Hama, Rural Damascus, Tartus, Hasakah, Raqqa, As-Suwayda, Daraa, and Deir ez-Zor.

Burnout was assessed using the Burnout Assessment Tool (BAT23), with scores categorized into three zones: Green (low), Orange (moderate), and Red (high).

Overall Burnout (BAT23): 50.0% of participants fell in the red zone, 21.5% in the orange, and 28.5% in the green.

Exhaustion: A high proportion (76.7%) were in the red zone.

Mental Disturbance: Most (66.7%) were in the green zone, indicating fewer symptoms.

Cognitive Impairment: Distribution was more even (38.7% green, 26.5% orange, 34.7% red).

Emotional Impairment: 48.5% were in the green zone, while 22.9% were in the red Table 2.

**Table 2:** Main results.

	Exhaustion	Mental disturbance	Cognitive impairment	Emotional Impairment	BAT23
Green (Low)	33 (6.0%)	359 (66.7%)	213 (38.7%)	267 (48.5%)	157 (28.5%)
Orange (Average)	95 (17.3%)	105 (19.5%)	146 (26.5%)	157 (28.5%)	118 (21.5%)
Red (high)	422 (76.7%)	74 (13.8%)	191 (34.7%)	126 (22.9%)	275 (50.0%)

Sex: No significant difference in BAT23 red zone prevalence between males (52.3%) and females (48.2%) (p = 0.634). However, males reported more emotional impairment (28.3% vs. 18.8%, p = 0.016).

Province: Although regional differences were noted (e.g., Idlib 58.7%, Damascus 58.4%, Aleppo 46.0%), they were not statistically significant (p = 0.108).

Marital Status: No significant differences in BAT23 (p = 0.424). Divorced individuals had 100% red-zone rates in all subcategories, but their sample size was small.

Residency Year: Burnout was more common in mid-level residents (years 2–4). Spearman’s rho showed a weak but significant negative correlation (r = −0.095, p = 0.025) hence burnout is more likely in young residents, but percentages tended to be higher in the middle years (years 2–4) compared to early or late years

Specialty: Strong associations were found (p < 0.0001). Internal medicine specialties had the highest BAT23 red zone rates (56.4%), followed by surgery (44.6%) and clinics (39.5%), in a more detailed look Pediatric surgery showed lower percentages of red zone in Cognitive Impairment and Emotional Impairment (27.3% each), whereas specialties like pulmonology, anesthesia, and neurology showed higher BAT23 red zones (above 78%).

Involvement in multiple centers: Residents working in more than one center had similar BAT23 distributions (p = 0.968). Hospital Type and Recruitment: No significant differences were found based on type of recruitment (p = 0.435) or hospital affiliation (p = 0.282) Table 3.

**Table 3:** Relationship between baseline characteristics and burnout.

		Ex Red	MD Red	CI Red	EI Red	BAT23 Red	Stat test	P-Value
Sex	Male	183 (77.2%)	37 (15.9%)	88 (37.1%)	67 (28.3%)*	124 (52.3%)	Chi-square	0.634
	Female	239 (76.4%)	37 (12.1%)	103 (32.9%)	59 (18.8%)*	151 (48.2%)		
Province	Aleppo	127 (72.2%)	19 (10.9%)	63 (35.8%)	40 (22.7%)	81 (46.0%)	Chi-square with Monte Carlo	0.108
	As-Suwayda	2 (100%)	0 (0%)	0 (0%)	0 (0%)	0 (0%)		
	Damascus	61 (79.2%)	12 (15.6%)	33 (42.9%)	18 (23.4%)	45 (58.4%)		
	Daraa	1 (100%)	0 (0%)	0 (0%)	1 (100%)	1 (100%)		
	Deir ez-Zor	1 (100%)	0 (0%)	0 (0%)	0 (0%)	0 (0%)		
	Hasakah	3 (100%)	0 (0%)	0 (0%)	0 (0%)	1 (33.3%)		
	Hama	4 (40%)	0 (0%)	2 (20.0%)	0 (0%)	3 (30.0%)		
	Homs	46 (75.4%)	9 (15.5%)	18 (29.5%)	14 (23.0%)	29 (47.5%)		
	Idlib	121 (84.6%)	26 (18.4%)	55 (38.5%)	41 (28.7%)	84 (58.7%)		
	Latakia	39 (69.6%)	6 (11.5%)	15 (26.8%)	9 (16.1%)	23 (41.1%)		
	Raqqa	2 (100%)	0 (0%)	1 (50%)	0 (0%)	1 (50%)		
	Rural Damascus	7 (77.8%)	2 (22.2%)	4 (44.4%)	3 (33.3%)	5 (55.6%)		
	Tartus	8 (88.9%)	0 (0%)	0 (0%)	0 (0%)	2 (22.2%)		
Marital status	Single	260 (74.9%)	45 (13.2%)	123 (35.4%)	75 (21.6%)	174 (50.1%)	Fisher’s exact	0.424
	Married	70 (76.1%)	14 (15.2%)	32 (34.8%)	26 (28.3%)	46 (50%)		
	Married with kids	88 (82.2%)	14 (13.7%)	32 (29.9%)	22 (20.6%)	51 (47.7%)		
	Divorced	4 (100%)	1 (25.0%)	4 (100%)	3 (75.0%)	4 (100%)		
Working in more than one center	Yes	107 (71.3%)	23 (15.6%)	55 (36.7%)	36 (24.0%)	74 (49.3%)	Chi-square	0.968
	No	315 (78.4%)	51 (13.0%)	136 (33.8%)	90 (22.4%)	201 (50.0%)		
Type of recruitment	Governmental	412 (76.9%)	74 (14.1%)	124 (23.1%)	188 (35.1%)	270 (50.4%)	Fisher’s exact	0.435
	Private	10 (71.4%)	0 (0%)	2 (14.3%)	3 (21.4%)	5 (35.7%)		
Type of Hospital	MOH	187 (78.9%)	39 (16.9%)*	64 (27.0%)	95 (40.1%)	121 (51.1%)	Chi-square	0.282
	MOHE	235 (75.1%)	35 (11.4%)*	62 (19.8%)	96 (30.7%)	154 (49.2%)		
Year of residency	0	1 (100%)	0 (0%)	1 (100%)	1 (100%)	1 (100%)	Spearman’s rho	0.025
	1	26 (74.3%)	6 (17.1%)	7 (20.0%)	10 (28.6%)	19 (54.3%)		
	2	160 (79.6%)	28 (14.4%)	55 (27.4%)	71 (35.3%)	107 (53.2%)		
	3	112 (75.2%)	18 (12.3%)	27 (18.1%)	54 (36.2%)	76 (51.0%)		
	4	100 (77.5%)	21 (16.7%)	33 (25.6%)	51 (39.5%)	63 (48.8%)		
	5	22 (64.7%)	1 (2.9%)	3 (8.8%)	4 (11.8%)	9 (26.5%)		
	6	1 (100%)	0 (0%)	0 (0%)	0 (0%)	0 (0%)		
Specialty (grouped)							Chi-square with Monte Carlo	<0.0001
*Internals*	Cardiology	19 (86.4%)	1 (4.5%)	6 (27.3%)	6 (27.3%)	7 (31.8%)		
	Endocrinology	8 (50.0%)	0 (0%)	1 (6.3%)	3 (18.8%)	5 (31.3%)		
	Family Medicine	8 (61.5%)	4 (30.8%)	3 (23.1%)	2 (15.4%)	6 (46.2%)		
	Gastroenterology	18 (90.0%)	1 (5.0%)	2 (10.0%)	7 (35.0%)	10 (50.0%)		
	General Internal Medicine	70 (83.3%)	15 (17.9%)	28 (33.3%)	34 (40.5%)	52 (61.9%)		
	Nephrology	9 (100%)	2 (22.2%)	2 (22.2%)	3 (33.3%)	6 (66.7%)		
	Neurology	10 (90.9%)	2 (18.2%)	3 (27.3%)	4 (36.4%)	9 (81.8%)		
	Pediatrics	71 (84.5%)	15 (18.3%)	22 (26.2%)	36 (42.9%)	47 (56.0%)		
	Pulmonology	18 (94.7%)	4 (22.2%)	8 (42.1%)	10 (52.6%)	15 (78.9%)		
	Rheumatology	5 (83.3%)	2 (33.3%)	1 (16.7%)	2 (33.3%)	3 (50.0%)		
*Surgeries*	Cardiac Surgery	3 (60.0%)	0 (0%)	1 (20.0%)	2 (40.0%)	2 (40.0%)		
	General Surgery	22 (75.9%)	2 (6.9%)	6 (20.7%)	10 (34.5%)	17 (58.6%)		
	Gynecology and Obstetrics	41 (63.1%)	2 (3.3%)	4 (6.2%)	10 (15.4%)	16 (24.6%)		
	Neurosurgery	6 (75.0%)	2 (25.0%)	2 (25.0%)	5 (62.5%)	5 (62.5%)		
	Ophthalmology	12 (60.0%)	0 (0%)	3 (15.0%)	5 (25.0%)	6 (30.0%)		
	Orthopedic Surgery	23 (82.1%)	5 (17.9%)	11 (39.3%)	12 (42.9%)	20 (71.4%)		
	Pediatric Surgery	8 (72.7%)	2 (20.0%)	3 (27.3%)	3 (27.3%)	4 (36.4%)		
	Urology	9 (81.8%)	2 (18.2%)	2 (18.2%)	5 (45.5%)	5 (45.5%)		
	Vascular Surgery	5 (71.4%)	1 (16.7%)	2 (28.6%)	4 (57.1%)	4 (57.1%)		
*Clinics*	Dermatology	1 (25.0%)	0 (0%)	0 (0%)	0 (0%)	0 (0%)		
	ENT	10 (71.4%)	1 (7.1%)	4 (28.6%)	6 (42.9%)	9 (64.3%)		
	Psychiatry	3 (42.9%)	0 (0%)	0 (0%)	2 (28.6%)	2 (28.6%)		
*Other*	Anesthesia and Resuscitation	11 (91.7%)	3 (27.3%)	4 (33.3%)	7 (58.3%)	11 (91.7%)		
	Oncology	3 (75.0%)	2 (50.0%)	2 (50.0%)	1 (25.0%)	2 (50.0%)		
	Pathology	8 (61.5%)	1 (7.7%)	1 (7.7%)	2 (15.4%)	2 (15.4%)		
	Radiology	6 (60.0%)	3 (30.0%)	3 (30.0%)	4 (40.0%)	4 (40.0%)		
	Other (collapsed)*	3 (60.0%)	1 (20.0%)	0 (0%)	1 (20.0%)	1 (20.0%)		
Type of specialty	Internals	242 (83.2%)*	48 (16.7%)	78 (26.8%)	110 (37.8%)*	164 (56.4%)	Chi-square	<0.0001
	Surgeries	118 (71.1%)*	16 (10.1%)	31 (18.7%)	52 (31.3%)*	74 (44.6%)		
	Clinics	23 (60.5%)*	1 (2.6%)	7 (18.4%)	11 (28.9%)*	15 (39.5%)		
	Other	39 (70.9%)*	9 (17.0%)	10 (18.2%)	18 (32.7%)*	22 (40.0%)		

Other (collapsed) includes: Hematology (n = 3), Thoracic Surgery (n = 1), Plastic Surgery (n = 1), Laboratory Medicine (n = 1)

The median age was similar across the three groups (Green, Orange, and Red), with a median of 27 years and no statistically significant difference (p = 0.96). However, the number of shifts worked in one month differed significantly between groups (p = 0.0011). The median number of shifts increased progressively from the green group (7 shifts, IQR 6) to the orange group (8 shifts, IQR 5), and was highest in the Red group (9 shifts, IQR 4), indicating a trend of Red > Orange > Green in terms of monthly workload Table 4.

**Table 4:** Age and number of shifts effect on burnout level.

	Green	Orange	Red	P value
Age (median, IQR)	27 (3)	27 (3)	27 (31)	0.96
Number of shift in one month (median, IQR)	7 (6)	8 (5)	9 (4)	0.0011

Significant differences were observed in both the ranking of the work environment and work satisfaction across the three groups (p < 0.0001 for both). The Green group reported the highest median ranking for work environment (6, IQR 2), followed by the orange group (5, IQR 8), and the red group reported the lowest (4, IQR 3). Similarly, work satisfaction rankings followed the same pattern, with the green group scoring highest (median 7, IQR 2), then Orange (median 6, IQR 2), and Red lowest (median 5, IQR 3) Table 5.

**Table 5:** Ranking work environment & satisfaction.

	Green	Orange	Red	P value
Ranking work environment (median, IQR)	6 (2)	5 (8)	4 (3)	<0.0001
Ranking work satisfaction (median, IQR)	7 (2)	6 (2)	5 (3)	<0.0001

Several factors related to burnout and stress were explored among participants across four burnout subcategories: Exhaustion (Ex), Mental Distance (MD), Cognitive Impairment (CI), Emotional Impairment (EI), in addition to the main BAT23 scores.

Dropping out and psychological consultation due to burnout showed highly significant associations with all burnout dimensions (p < 0.0001). A notably high percentage of individuals in the Exhaustion Red group reported considering dropping out (94.1%) and seeking psychological consultation (87.6%).

Ways to destress varied, with social support being the most reported method (75.6%), followed by walking (70.7%). Although these trends were observed, the association with burnout dimensions did not reach statistical significance (p = 0.056).

Experiencing improper treatment showed strong associations across all burnout dimensions (p < 0.0001), with 85.5% of those in the Exhaustion red group reporting improper treatment noting that improper treatment isn’t significantly related to year of residency (Fisher’s p = 0.534)

A lack of time for family and friends, poor training, and work overload were all significantly associated with higher burnout scores (p < 0.0001). For example, only 56.6% of Exhausted Red participants felt they had time for family and friends, and just 69.1% felt they were receiving decent training.

The perceived fairness in workload distribution was also linked to burnout (p = 0.044), with lower agreement among high burnout subgroups.

Migration due to work conditions was significantly associated with burnout (p = 0.034), with 80.4% of those in Exhaustion red zone considering migration.

Regarding factors affecting stress, workload, lack of support, and work environment were most strongly linked to burnout dimensions, all showing statistical significance (p < 0.05), particularly workload and lack of support (p < 0.0001). Other factors like administrative tasks were also associated (p = 0.038).

Finally, days off per year were inversely related to burnout, although the correlation was weak (Spearman’s rho = −0.057, p = 0.181) for the total BAT score, however those reporting fewer days off showed higher cognitive impairment levels (Spearman’s rho = −0.126 p = 0.003) Table 6.

**Table 6:** Association of Burnout Dimensions with Work-Related Factors, Coping Strategies, and Wellbeing Indicators.

		Ex Red	MD Red	CI Red	EI Red	BAT23 Red	Stat test	P-Value
Ways to destress	Social support	127 (75.6%)	25 (15.3%)*	60 (35.7%)	44 (26.2%)*	78 (46.4%)	Chi-square	0.056
	Walking	135 (70.7%)	14 (7.6%)*	61 (31.9%)	31 (16.2%)*	85 (44.5%)		
	Sports	39 (79.6%)	6 (12.5%)*	19 (38.8%)	11 (22.4%(*	29 (59.2%)		
	Other	121 (85.2%)	29 (20.4%)*	51 (35.9%)	40 (28.2%)*	83 (58.5%)		
Dropping out due to Burn out		48 (94.1%)*	26 (28.5%)*	21 (36.2%)*	38 (55.9%)*	83 (79.8%)	Chi-square	<0.0001
Psychologic consultation due to Burn out		45 (87.6%)*	17 (18.4%)*	20 (35.2%)*	34 (50.3%)*	72 (69.0%)	Chi-square	<0.0001
Shortage of medical equipments		39 (77.1%)	13 (14.1%)	14 (24.3%)*	25 (35.8%)*	53 (50.9%)	Chi-square	0.426
Work is evenly distributed		36 (70.9%)*	13 (14.1%)	11 (18.3%)	22 (31.4%)	44 (42.3%)	Chi-square	0.044
Have been bullied		43 (85.5%)*	15 (16.7%)*	17 (29.7%)*	30 (42.1%)*	64 (61.0%)	Chi-square	<0.0001
Wage covers basic needs		39 (78.0%)	7 (8.2%)	9 (16.0%)	16 (22.0%)	80 (55.6%)	Chi-square	0.472
Time for Family and Friends		28 (56.6%)*	8 (9.4%)	9 (15.9%)*	15 (21.2%)*	32 (31.0%)	Chi-square	<0.0001
Getting decent training		34 (69.1%)	4 (4.2%)*	8 (13.8%)	19 (27.6%)*	34 (32.5%)	Chi-square	<0.0001
Migrating due to work circumstances		41 (80.4%)*	13 (14.6%)*	15 (26.1%)*	27 (39.1%)	57 (54.7%)	Chi-square	0.034
Factors affecting stress	Workload	42 (82.5%)*	12 (13.7%)	16 (26.7%)*	58 (56.3%)*	213 (56.3%)	Chi-square	<0.0001
	Patient caring	40 (81.9%)	13 (15.5%)*	128 (31.5%(*	57 (55.6%)	80 (55.6%)	Chi-square	0.092
	Administration tasks	39 (80.1%)	13 (14.9%)*	17 (27.4%)	57 (55.6%)*	148 (55.6%)	Chi-square	0.038
	Work environment	293 (82.3%)*	47 (13.4%)	51 (26.3%)*	94 (26.4%)*	100 (52.4%)	Chi-square	0.002
	Lack of support	293 (82.3%)*	47 (13.4%)*	102 (41.8%)*	94 (26.4%)*	197 (55.3%)	Chi-square	<0.0001
	other	71 (75.5%)	13 (14.0%)	32 (34%)	23 (24.5%)	51 (54.3%)	Chi-square	0.075
Days off (yearly)	0-5 days	142 (76.8%)	31 (17.0%)	50 (27.0%)*	80 (43.2%)	104 (56.2%)	Spearman’s rho	0.181
	6-10 days	89 (80.2%)	12 (11.2%)	26 (23.4%)*	41 (36.9%)	56 (50.5%)		
	1-15 days	135 (75.0%)	22 (12.5%)	34 (18.9%)*	50 (27.8%)	81 (45.0%)		
	More than 15 days	56 (75.7%)	9 (12.3%)	16 (21.6%)*	20 (27.0%)	34 (45.9%)		

## Discussion

Prolonged exposure to war has been associated with mental health. Physicians in crisis-affected regions may face elevated risks of stress conditions, like Burnout, due to healthcare system collapse, economic instability, resource shortages, and overwhelming patient loads identified as contributing factors in the literature**.**

In our study, the BAT was selected for comprehensive clinical assessment of burnout, integrating core dimensions (exhaustion, cognitive/emotional impairment, mental distance) and secondary manifestations (psychological distress, psychosomatic complaints). This contrasts with the Maslach Burnout Inventory (MBI), historically the dominant tool operationalizing burnout through three subscales (emotional exhaustion, depersonalization, reduced efficacy). [11,12] While the MBI has been widely used for decades in occupational and psychological research, its fragmented structure, lack of a unified diagnostic score, and exclusion of critical cognitive/emotional deficits limited its clinical utility [10].

The BAT was employed to overcome these limitations, offering a syndromic framework with validated psychometric robustness for precise evaluation of burnout severity and progression.

Although the questionnaire did not ask about individual experiences of violence or displacement, the national-level evidence of facility damage, workforce exodus, and humanitarian demand described above [7–9] shows that the remaining residents now practice within a collapsing infrastructure. We therefore relate our findings to the systemic stressors of the conflict rather than to direct exposures for each respondent.

Our study identified a comparable prevalence of physician burnout (50.0%) to that reported by Shenoi et al. among U.S. physicians (49.0%) [13]. However, this numerical equivalence requires careful interpretation, as the two studies employed fundamentally different measurement tools with distinct conceptual frameworks. Shenoi et al. used the Maslach Burnout Inventory (MBI), which operationalizes burnout through three subscales—emotional exhaustion, depersonalization, and reduced personal accomplishment—and classifies burnout as scoring high on at least one subscale [13]. In contrast, our study employed the Burnout Assessment Tool (BAT-23), a more recent instrument that conceptualizes burnout as a syndrome comprising four core dimensions (exhaustion, mental distance, cognitive impairment, and emotional impairment) and provides a unified composite score for determining burnout risk. The BAT was specifically designed to address limitations of the MBI, including its fragmented structure and lack of a unified diagnostic score [10].

Consequently, while the headline prevalence rates appear similar, the underlying constructs and thresholds for classification differ substantially. Notably, the rate of severe burnout in the Shenoi et al. study—defined as high emotional exhaustion combined with high depersonalization or low personal accomplishment—was 21%, whereas 50% of our participants fell into the highest-risk (Red) category on the BAT-23 composite score. This discrepancy underscores that direct cross-study comparisons of burnout prevalence are methodologically challenging when different instruments are used.

Beyond measurement differences, the numerical similarity obscures profound disparities in underlying drivers, systemic contexts, and clinical implications. The U.S. study identified regular physical exercise as protective and noted Sex differences in burnout risk, but did not find strong associations with organizational factors [13]. In contrast, our findings in Syria reveal burnout tightly linked to systemic healthcare collapse, resource scarcity, excessive workloads, and inadequate training—factors shaped by over a decade of conflict, workforce attrition, and infrastructural deterioration. Thus, while the prevalence figures may appear equivalent, the etiologies, manifestations, and potential solutions for burnout in these two contexts are likely vastly different.

The burnout profile revealed striking disparities across subdomains. Exhaustion emerged as the most severe dimension, with 76.7% of residents in the red zone (high risk)—a finding that reflects extreme physical and mental depletion. In contrast, mental disturbance showed relative resilience; 66.7% of participants were in the green zone (low risk), suggesting preserved psychological stability among most residents. Cognitive impairment displayed a tripartite distribution: 38.7% (green), 26.5% (orange), and 34.7% (red), indicating moderate-to-severe deficits in memory, focus, or decision-making for over 60% of residents. Emotional impairment was predominantly green (48.5%), though 22.9% fell into the red zone, highlighting vulnerabilities in emotional regulation among a subset.

### Contributing factors to burnout

- **Work Overload and Resource Scarcity**

Collectively, these BAT23-derived findings underscore exhaustion as the central driver of burnout, with systemic stressors overwhelming residents’ physical and cognitive capacities, even as baseline mental health remains relatively intact. This dissociation suggests that interventions targeting workload reduction and recovery time may be important targets for future intervention studies, potentially complementing psychological support approaches.

Our study identified divergent temporal patterns of burnout severity compared to Bahaa et al. (2019). [3] While Bahaa et al. reported escalating burnout levels culminating in peak prevalence during the final residency years, our findings demonstrate a distinct shift, with burnout peaking significantly earlier during mid-level residency (Years 2–4).

The elevated burnout prevalence among mid-level residents (Years 2–4) was observed in the context of Syria’s systemic attrition of senior physicians through mass migration and the subsequent collapse of clinical mentorship structures. With experienced specialists leaving the country, mid-level trainees reported assuming unsupervised roles—managing complex cases, leading procedures, and making high-stakes decisions—despite lacking adequate training or institutional support. This role incongruity, combined with the absence of hierarchical guidance in specialties like surgery and internal medicine, was associated with increased workload burdens and ethical dilemmas, accelerating burnout during these transitional years. Whereas a study of Lebanese residents who were 2.91 more likely to improve burnout due to prolonged work hours of more than 80 h/per week than residents who work less than this regularly [9].

Reports examining the relationship between age, career duration, and burnout have shown variable results. However**,** overall findings suggest that the relationship between age and burnout follows a bimodal pattern, where both younger individuals (under 25) and older individuals (over 55), along with those with greater work experience, tend to experience higher levels of burnout. Many studies indicate that younger age is a significant predictor of burnout. For instance, a study conducted among physicians in Saudi Arabia (N = 270) found that younger age was significantly associated with higher levels of emotional exhaustion and lower scores in personal accomplishment [5,10].

- **Burnout and Specialties**

Furthermore, our analysis revealed a robust statistical association between medical specialty and burnout risk (p < 0.0001), with disproportionately high rates observed in internal medicine (56.4%) and surgical specialties (44.6%). Notably, subspecialties such as pulmonology, anesthesia, and neurology exhibited extreme burnout prevalence (>78%), underscoring domain-specific vulnerabilities. In contrast, Bahaa et al. (2019) found no statistically significant correlation between specialty and burnout severity, though they descriptively noted elevated emotional exhaustion in general surgery and emergency medicine (89%).

Specialties such as anesthesiology and pulmonology showed disproportionately high burnout in association with their high-stakes, resource-dependent workflows and Syria’s systemic healthcare collapse. In anesthesiology, residents reported face relentless exposure to surgical emergencies requiring precision in critical care, along with shortages of essential supplies (e.g., anesthetics, monitoring devices). For pulmonology, the COVID-19 pandemic coincided with stressors, as residents reported managing acute respiratory failures amid severe oxygen and ventilator shortages. These specialties inherently involve ethical dilemmas (e.g., triaging care during equipment scarcity) and frequent exposure to patient mortality, which have been linked to moral injury and emotional exhaustion in the literature. The absence of institutional safeguards—such as structured debriefing or psychological support—may contribute to these pressures, potentially exacerbating the high burnout rates observed.

In a study of anesthesiologists in Egypt, researchers found a significant positive correlation between job stress, measured by the Workplace Stress Scale of the American Institute of Stress, and the subscales of the Maslach Burnout Inventory – Human Services Survey (MBI-HSS). [5] Additionally, among oncology nurses in Turkey, there was a significant positive relationship between the subdimensions of job stress, evaluated using the Job Stressors Scale, and the level of burnout (p < 0.05) [14]. Furthermore, among the anesthesiologists in Egypt (N = 98), the lack of job supports emerged as the strongest single predictor of all burnout dimensions [15].

- **Burnout and Sex**

Research in the Middle East supports earlier findings among healthcare providers, showing that key risk factors for burnout include being female, younger age, and having insufficient support and resources to manage workload [16]. Similarly, in our study younger age was more likely to correlate with higher risk of burnout, whereas males reported more emotional impairment.

- **Impact of Days-off on physicians’ mental health**

The minimal impact of leave days on reducing burnout underscores the deep-seated systemic issues plaguing Syria’s healthcare sector, where insufficient leave policies (such as a median of just 7 days per year for high-burnout groups) fail to address the relentless stressors caused by unmanageable workloads—compounded by the fact that residents often remain on-call due to severe staffing shortages. Structural inequities, like poverty-level salaries forcing workers to take on extra shifts at multiple hospitals, further undermine rest periods, while institutional stigma discourages leave usage by framing it as a sign of professional weakness.

Consequently, leave becomes a nominal respite rather than a restorative measure, as residents re-enter unchanged, high-stress environments with unaddressed systemic drivers.

These discrepancies likely stem from multifactorial systemic collapses unique to the post-2019 period in Syria. The compounding effects of the COVID-19 pandemic, Caesar Act sanctions (which crippled medical supply chains and funding), and inequitable health policies under the Syrian regime precipitated a near-total collapse of healthcare infrastructure. This collapse disproportionately intensified clinical workloads, training deficiencies, and psychological strain on residents, particularly in high-volume specialties like internal medicine and surgery.

The pandemic overwhelmed understaffed hospitals, forcing mid-level residents (Years 2–4) to assume roles beyond their training level, while sanctions restricted access to essential equipment and medications, amplifying procedural and ethical stressors. Concurrently, the regime’s prioritization of militarized healthcare over public institutions eroded institutional support, exacerbating burnout in specialties reliant on systemic resources (e.g., pulmonology, anesthesia).

### Interventions aiming to overcome burnout

There is a critical shortage of programs aimed at addressing burnout in the Middle East, with only a few studies exploring interventions.

In Qatar, Ghannam et al. analyzed a stress management workshop for 256 residents. This one-day event taught participants to identify stressors and utilize intervention techniques. Remarkably, one month later, 83.6% reported applying new skills, with significant improvements noted in emotional exhaustion, depersonalization, and overall satisfaction with their medical practice [17].

Additionally, Kotb et al. assessed an educational program for 31 family physicians in Egypt, finding a significant decrease in burnout prevalence from 41.9% to 32.3% after six months [18].

These findings suggest that further research and systematic efforts to address burnout among healthcare providers in the Middle East are warranted. While this study was not designed to evaluate interventions, the factors associated with burnout—including excessive workloads, insufficient training, lack of institutional support, and challenging work environments—suggest several areas that warrant consideration in future policy planning and interventional research.

Potential strategies that merit exploration include: (1) evaluating whether increased annual leave with adequate staffing coverage may reduce burnout, noting that in this study leave days alone were not significantly associated with burnout severity; (2) testing workload reduction through equitable task distribution and implementation of mental health support programs; (3) addressing staff shortages through targeted hiring and incentives for high-need areas; (4) assessing the impact of improved base salaries on reducing reliance on supplemental shifts; (5) developing institutional policies to address stigma around leave-taking; and (6) establishing monitoring systems to track burnout and evaluate intervention effectiveness.

Future longitudinal and interventional studies are needed to determine which of these approaches are effective in the Syrian context. Any such reforms would require coordinated efforts among government, hospital administrations, and international health organizations to ensure sustainable implementation.

This study has limitations. Its cross-sectional design and self-reported measures preclude causal inference, and the recruitment strategy around specific governorate rosters introduces selection bias. We did not capture individual-level exposures to violence or displacement, so our linkage of burnout patterns to the conflict remains contextualized at the national level rather than based on respondent-specific trauma. The modest counts in some specialty and province categories also limit the stability of potential multivariable models, which is why we present descriptive and bivariate analyses and encourage future work with larger, longitudinal cohorts to implement regression-based adjustment for confounding.

Additionally, we acknowledge the limitation of relying primarily on bivariate statistical methods (Chi-square, Kruskal-Wallis, and Spearman correlations) rather than multivariable regression modeling. While this approach adequately describes the prevalence and associations of burnout within this specific sample, it does not provide adjusted risk measures (Odds Ratios) to control for confounding variables. Due to the modest sample size and the fragmentation of data across numerous specialty and provincial subgroups—which resulted in small cell counts and model instability when logistic regression was attempted—we elected to present the more robust bivariate findings. Future studies with larger, pooled cohorts are necessary to perform multivariate regression to identify independent predictors of burnout in this population.

## Conclusion

This study reveals a concerning prevalence of burnout among Syrian medical residents, with 50% demonstrating severe symptoms, particularly in exhaustion and cognitive impairment. The highest rates were observed among mid-level trainees and those in high-intensity specialties, consistent with the compounded impact of systemic healthcare collapse, excessive workloads, and inadequate training. These findings highlight potential targets for structural reforms, including workload redistribution, improved training, and enhanced mental health support. Future research is needed to evaluate whether such interventions may reduce burnout and support the sustainability of Syria’s healthcare system. To our knowledge, this is the first multi-governorate application of the Burnout Assessment Tool within Syria’s residency programs, offering a comprehensive baseline of exhaustion, cognitive, and emotional domains to inform monitoring and intervention planning moving forward.

## Supporting information

S1 File Minimal anonymized dataset. De-identified participant-level dataset used for all analyses in this study, including demographic variables, training and work-related characteristics, Burnout Assessment Tool (BAT-23) subscale and total scores, burnout risk categories, and all variables used to generate Tables 1–6.
(XLSX)

S2 File Data dictionary. Codebook describing all variables included in the minimal dataset, including variable names, definitions, and coding schemes for categorical responses and burnout classifications.
(DOCX)

## Abbreviations

BAT: Burnout Assessment Tool
MOHE: Ministry of Higher Education
MOH: Ministry of Health
IQR: : interquartile range [IQR]
EX: Exhaustion
MD: Mental Distance
CI: Cognitive Impairment
EI: Emotional Impairment

## References

[1] Freudenberger HJ. Staff Burn-Out. Journal of Social Issues. 1974;30(1):159–65. doi: 10.1111/J.1540-4560.1974.TB00706.X

[2] Burn-out an “occupational phenomenon”: International Classification of Diseases. https://www.who.int/news/item/28-05-2019-burn-out-an-occupational-phenomenon-international-classification-of-diseases. Accessed 2025 May 23.

[3] Alhaffar BA, Abbas G, Alhaffar AA. The prevalence of burnout syndrome among resident physicians in Syria. J Occup Med Toxicol. 2019;14(1). doi: 10.1186/S12995-019-0250-0 PMC6902600 31827575

[4] Rodrigues H, Cobucci R, Oliveira A, Cabral JV, Medeiros L, Gurgel K, et al. Burnout syndrome among medical residents: A systematic review and meta-analysis. PLoS One. 2018;13(11):e0206840. doi: 10.1371/journal.pone.0206840 30418984 PMC6231624

[5] Rothenberger DA. Physician burnout and well-being: A systematic review and framework for action. Dis Colon Rectum. 2017;60(6):567–76. doi: 10.1097/DCR.0000000000000844 28481850

[6] Salvagioni DAJ, Melanda FN, Mesas AE, González AD, Gabani FL, Andrade SM. Physical, psychological and occupational consequences of job burnout: A systematic review of prospective studies. PLoS One. 2017;12(10):e0185781. doi: 10.1371/journal.pone.0185781 28977041 PMC5627926

[7] WHO calls for urgent support to rebuild Syria’s health system. https://www.who.int/news/item/2025-urgent-support-syria-health-system. 2025.

[8] WHO urgent flash appeal for the health emergency response in Syria: US$ 56.4 million for critical needs on multiple fronts. 2024.

[9] Donors’ support for WHO work in Syria makes a difference in protecting and improving health in the country. 2025.

[10] Schaufeli WB, Desart S, De Witte H. Burnout Assessment Tool (BAT)—Development, Validity, and Reliability. International Journal of Environmental Research and Public Health. 2020;17(24):9495. doi: 10.3390/IJERPH17249495 33352940 PMC7766078

[11] Maslach C, Schaufeli WB, Leiter MP. Job burnout. Annu Rev Psychol. 2001;52:397–422. doi: 10.1146/annurev.psych.52.1.397 11148311

[12] Tomás JM, de Los Santos S, Alonso-Andres A, Fernández I. Validation of the maslach burnout inventory-general survey on a representative sample of dominican teachers: Normative data. Span J Psychol. 2016;19:E83. doi: 10.1017/sjp.2016.91 27873562

[13] Shenoi AN, Kalyanaraman M, Pillai A, Raghava PS, Day S. Burnout and psychological distress among pediatric critical care physicians in the United States. Crit Care Med. 2018;46(1):116–22. doi: 10.1097/CCM.0000000000002751 29016364

[14] Tuna R, Baykal Ü. The relationship between job stress and burnout levels of oncology nurses. Asia Pac J Oncol Nurs. 2014;1(1):33–9. doi: 10.4103/2347-5625.135818 27981080 PMC5123449

[15] Shams T, El-Masry R. Job stress and burnout among academic career anaesthesiologists at an Egyptian university hospital. Sultan Qaboos Univ Med J. 2013;13(2):287–95. doi: 10.12816/0003236 23862036 PMC3706120

[16] Elbarazi I, Loney T, Yousef S, Elias A. Prevalence of and factors associated with burnout among health care professionals in Arab countries: A systematic review. BMC Health Serv Res. 2017;17(1):491. doi: 10.1186/s12913-017-2319-8 28716142 PMC5513024

[17] Ghannam J, Afana A, Ho EY, Al-Khal A, Bylund CL. The impact of a stress management intervention on medical residents’ stress and burnout. International Journal of Stress Management. 2020;27(1):65–73. doi: 10.1037/str0000125

[18] Kotb AA, Mohamed KA-E, Kamel MH, Ismail MAR, Abdulmajeed AA. Comparison of burnout pattern between hospital physicians and family physicians working in Suez Canal University Hospitals. Pan Afr Med J. 2014;18:164. doi: 10.11604/pamj.2014.18.164.3355 25422682 PMC4239452

