# Supplementary material for: Burnout among Syrian medical residents: A cross-sectional study using the burnout assessment tool (BAT)
Source: PLoS One. 2026 Jun 15;21(6):e0350426. doi: 10.1371/journal.pone.0350426 (PMC13268169; doi:10.1371/journal.pone.0350426)
Supplement: S2 File — Codebook describing all variables included in the minimal dataset, including variable names, definitions, and coding schemes for categorical responses and burnout classifications. (DOCX) [file pone.0350426.s002.docx]

| Variable name | Description | Coding |
| --- | --- | --- |
| yes_no | Binary response variable | 0 = No, 1 = Yes |
| specialty | Medical specialty of the resident | 0 = Pediatric surgery, 1 = General internal medicine, 2 = Neurology (internal), 3 = General surgery, 4 = Orthopedic surgery, 5 = Pathology, 6 = Pediatrics, 7 = Vascular surgery, 8 = Pulmonology (chest), 9 = Musculoskeletal medicine, 10 = Ophthalmology, 11 = Radiology and medical imaging, 12 = Endocrinology, 13 = Gynecology and obstetrics, 14 = Anesthesia and resuscitation, 15 = ENT, 16 = Dermatology, 17 = Nephrology, 18 = Neurosurgery, 19 = Hematology, 20 = Family medicine, 21 = Oncology, 22 = Cardiac surgery, 23 = Gastroenterology, 24 = Thoracic surgery, 25 = Urology, 26 = Plastic surgery, 27 = Cardiology, 28 = Laboratory medicine, 29 = Psychiatry, 30 = Other |
| hospital_sector | Type of recruiting hospital | 0 = Private hospital, 1 = Governmental hospital |
| gender | Participant gender | 0 = Female, 1 = Male |
| hospital_affiliation | Hospital affiliation | 0 = Ministry of Health (MOH), 1 = Ministry of Higher Education (MOHE) |
| marital_status | Marital status | 0 = Single, 1 = Married, 2 = Married with children, 3 = Divorced |
| way_to_destress | Main way to relieve stress | 0 = Social support, 1 = Other, 2 = Walking / leisure, 3 = Sports |
| province | Governorate of training | 0 = Aleppo, 1 = Homs, 2 = Damascus, 3 = Hama, 4 = Daraa, 5 = Rural Damascus, 6 = Hasakah, 7 = Latakia, 8 = Idlib, 9 = Tartus, 10 = Deir ez-Zor, 11 = As-Suwayda, 12 = Raqqa |
| days_off_category | Annual days off category | 0 = 0–5 days, 1 = 6–10 days, 2 = 11–15 days, 3 = More than 15 days |
| specialty_group | Main specialty group | 0 = Internals, 1 = Surgeries, 2 = Clinics, 3 = Other |
| bat_zone | Overall BAT-23 burnout risk category | 0 = Green (low risk), 1 = Orange (moderate risk), 2 = Red (high risk) |
