# Supplementary material for: Burnout among Syrian medical residents: A cross-sectional study using the burnout assessment tool (BAT)
Source: PLoS One. 2026 Jun 15;21(6):e0350426. doi: 10.1371/journal.pone.0350426 (PMC13268169; doi:10.1371/journal.pone.0350426)
Supplement: Attachment — Submitted filename: reply to reviewer.docx [file pone.0350426.s004.docx]

Dear Dr. Hendy and reviewers,

Thank you very much for the constructive feedback on PONE-D-25-62647. We believe the manuscript is substantially stronger after revising the introduction, methods, results, and discussion to address the points raised below. The revised submission now includes the requested response letter, a tracked-changes version, a clean copy, updated supporting information captions, and a clarified data availability statement with the dataset provided directly.

**Reviewer 1**

Linking burnout to the Syrian crisis context. We expanded the Introduction and Discussion to cite recent literature describing how protracted conflict, sanctions, and health-system collapse elevate burnout risk, and we explicitly frame our findings within that broader context while acknowledging that our study assesses symptoms rather than exposure to war per se.

- Comparison with other settings. The Discussion now contrasts our 50% BAT red-zone prevalence with both regional and international studies (e.g., Shenoi et al., Bahaa et al.) to clarify where our figures align or diverge and why Syria’s systemic stressors may explain any similarities.
- Response rate. The Methods and Results sections now report the full sampling frame (residents invited via the six governorate registries) and the response rate, emphasizing the representativeness of the cohort.
- Clarify that the survey targeted residents. We have revised the Methods to state that only physicians enrolled in residency programs (postgraduate training years 1+) were eligible, and we removed language that could be interpreted as including staff physicians.
- Definition of “clinics.” We now explain that the “clinical” category covers non-surgical subspecialties such as dermatology, psychiatry, and rehabilitation medicine to differentiate it from purely internal medicine or surgical tracks.
- Province table organization. The province breakdown is now presented once, sorted by prevalence (highest to lowest red-zone rates) with each governorate clearly labeled, and the redundant listing has been removed.
- Multivariable analysis. Because several categorical predictors had sparse cells (especially when subdivided by specialty and province), multivariable regression produced unstable estimates, so we instead added stratified comparisons and emphasized the most robust associations (e.g., internal medicine, surgery, pulmonology/ anesthesia/neurology) while clearly describing the limitations of further adjustment.
- Detailing which categories drive significance. The Results now include descriptive text identifying which specialties, residency years, and workplace factors correspond to the elevated p-values (e.g., internal medicine specialty red-zone rate = 56.4%, mid-level residents have the highest proportion of exhaustion red zone, etc.), and we highlight that marital status did not differ meaningfully across domains.
- Manuscript flow and war exposure language. We smoothed paragraph transitions throughout the Results and Discussion and softened statements that previously linked burnout directly to war exposure, replacing them with phrasing such as “amid the protracted crisis” and noting that we infer broader systemic pressures rather than having measured each stressor.

**Reviewer 2**

- IRB number consistency. The ethics section in Methods now consistently cites Approval No. 2527, and the standalone ethics statement has been moved entirely into Methods, eliminating the duplicate numbering.
- Data availability. The manuscript no longer states “data available upon request”; instead, All data generated or analysed during this study are included in this published article [and its supplementary information files].
- Reduce repetitive numbers. We trimmed redundant numeric reporting in the Results and Discussion, focusing instead on the most informative comparisons (e.g., we report the median number of shifts once with the associated p-value rather than repeating it across sections).
- Highlight what makes the study unique. The Conclusion now states that this is the first BAT-based nationwide assessment of Syrian residents (across six governorates) that highlights mid-level trainees and high-intensity specialties, underscoring the novel contribution.
- Grammar and language. We proofread the manuscript and corrected grammar issues throughout, particularly in the Discussion and Conclusion.
- Limitations. A limitations paragraph now appears near the end of the Discussion, acknowledging the cross-sectional design, self-reported measures, and the challenges of modeling complex work-environment relationships.

**Reviewer 3**

- Reference list balance. We expanded the literature cited to include additional recent work on burnout in conflict-affected and resource-constrained settings (e.g., regional cohort studies and systematic reviews) to achieve a more comprehensive and unbiased reference list.
- Critical view of the research area. The Discussion now reflects this broader perspective by acknowledging both the strengths of BAT and the ongoing debates about measuring burnout, thereby aligning with the reviewer’s request for a balanced appraisal.

We hope these revisions satisfactorily address the reviewers’ concerns. The revised manuscript, tracked changes, and supporting documents have been uploaded in accordance with PLOS ONE’s submission requirements.
