# Supplementary material for: Burnout among Syrian medical residents: A cross-sectional study using the burnout assessment tool (BAT)
Source: PLoS One. 2026 Jun 15;21(6):e0350426. doi: 10.1371/journal.pone.0350426 (PMC13268169; doi:10.1371/journal.pone.0350426)
Supplement: Attachment — Submitted filename: Response to Reviewers.docx [file pone.0350426.s005.docx]

**Response to Reviewers**

We thank the reviewers for their thoughtful and constructive comments, which have significantly improved the quality of this manuscript. We have carefully addressed each concern raised. Below we provide a point-by-point response to all reviewer comments.

**Response to Reviewer #1**

| Comment | Response |
| --- | --- |
| 1. Table 1: The specialty order remains unclear. Why is it ordered like that? Would it make more sense to list the specialty types and then under each type, list the specialties attributed to that type, and list them either in alphabetical order or order of frequency? Also, Table 1 has repeated all the data twice. | We thank the reviewer for this observation. The duplicate Table 1 has been removed. Additionally, we have reorganized the specialty list in Table 1 into logical groups (*Internals*, *Surgeries*, *Clinics*, *Other*) with specialties listed alphabetically within each group for clarity and ease of reference. |
| 2. The discussion still has comments that sound like direct causation. Please review in detail as that language likely needs to be softened or backed up with references in the literature. | We have thoroughly revised the Discussion section to replace causal language with associative language throughout. Key changes include: "is attributable to" → "was observed in the context of"; "exacerbated" → "was associated with"; "due to" → "in association with"; "fuels" → "has been linked to"; "highlights the urgent need" → "suggests that further research is warranted." All mechanistic claims have been reframed to reflect the cross-sectional design of the study. |
| 3. The paper states burnout is the same as a study from the US but the underlying metrics are different. I think this needs to be expanded on as it's a key point. | We have expanded this section substantially. We now explicitly compare the Burnout Assessment Tool (BAT) used in our study with the Maslach Burnout Inventory (MBI) used by Shenoi et al., highlighting differences in conceptual frameworks (4 dimensions vs. 3 subscales), scoring methods (unified composite score vs. high in ≥1 subscale), and severe burnout rates (50% in our study vs. 21% in Shenoi et al.). We emphasize that direct cross-study comparisons are methodologically challenging and that the numerical similarity in prevalence obscures profound differences in context and drivers. |
| 4. The discussion suggests measures to mitigate burnout, but this was not the focus of the paper and there is no clear data you raise to say these things will bring improvement. For instance, you first state there is no association with leave days and burnout but then suggest more leave days. | We have revised the recommendations section to address this concern. The measures are now framed as "potential strategies that merit exploration" rather than definitive solutions. We explicitly note that leave days alone were not significantly associated with burnout severity in this study (rho = -0.057, p = 0.181) and that future interventional studies are needed to evaluate efficacy. The section now emphasizes that these are areas warranting consideration in future research, not conclusions derived from our data. |
| 5. Please review grammar and language use as the discussion remains challenging to follow at times. | We have conducted a comprehensive grammar and language review. Overly long sentences have been broken into shorter, clearer statements; transitions have been added where missing; run-on sentences have been split; and redundant phrasing has been removed. The Discussion section now reads more smoothly. |

**Response to Reviewer #2**

| Comment | Response |
| --- | --- |
| 1. The Methods section cites IRB Approval No. 2527, while the Ethics section cites No. 2408. | We thank the reviewer for identifying this discrepancy. We have verified that the correct approval number is 2527. The Ethics section has been revised to reflect this number consistently throughout the manuscript. |
| 2. Survey distribution method is not described, no response rate is reported, and the total number of eligible residents contacted is never stated. Without this, the sample's representativeness cannot be assessed. | We have substantially expanded the Methods section to address this concern. We now clearly describe: (1) the sampling frame (620 eligible residents from master lists obtained from program coordinators); (2) the multi-channel distribution method (institutional email, WhatsApp groups, direct follow-up); (3) the reminder schedule (days 3, 7, and 10); and (4) the response rate (550/620 = 88.7%). The high response rate supports the representativeness of the sample. |
| 3. Type of recruitment rows in Table 3 appear to have the Gov/Private labels transposed, contradicting the baseline data. | We thank the reviewer for this careful observation. The labels have been corrected in Table 3. The Governmental row now correctly shows n=412 (76.9%) and the Private row shows n=10 (71.4%), consistent with baseline data in Table 1. |
| 4. Specialties with n<5 (e.g., Thoracic Surgery n=1, Plastic Surgery n=1) are included in inferential testing. These should be collapsed into "other" or excluded from statistical comparisons. | We have addressed this by collapsing specialties with n<5 (Hematology, Thoracic Surgery, Plastic Surgery, Laboratory Medicine) into an "Other (collapsed)" category in Table 3. The statistical comparisons (chi-square with Monte Carlo simulation) now respect the assumptions of the test, and the p-value (<0.0001) remains valid. |
| 5. All mechanistic claims must be reframed using associative language throughout. | As noted in response to Reviewer #1, we have comprehensively revised the Discussion section to replace causal language with associative language. All claims now reflect associations rather than causal relationships, consistent with the cross-sectional study design. |

**Response to Reviewer #3**

| Comment | Response |
| --- | --- |
| This paper has a good quality for writing but the references list doesn't cover the relevant literature adequately and in an unbiased manner. The reference list must be improved. The research should include a balanced, comprehensive, and critical view of the research area. | We thank the reviewer for this constructive feedback. We have substantially improved the reference list by: (1) adding the Maslach Burnout Inventory references (Maslach & Jackson, 1986; Maslach et al., 2001) for proper context when comparing with the BAT; (2) including Shenoi et al. (2018) as the US comparison study; (3) adding a Middle East systematic review (Elbarazi et al., 2017) to provide regional context; (4) completing the incomplete citation for Tuna and Baykal (oncology nurses); (5) ensuring all references are formatted consistently with DOIs where available.  The reference list now provides a balanced and comprehensive view of the literature. |

We are grateful for the reviewers' time and expertise, which have helped strengthen this manuscript. We hope the revised version is now suitable for publication.
