# Supplementary material for: Burnout among Syrian medical residents: A cross-sectional study using the burnout assessment tool (BAT)
Source: PLoS One. 2026 Jun 15;21(6):e0350426. doi: 10.1371/journal.pone.0350426 (PMC13268169; doi:10.1371/journal.pone.0350426)
Supplement: Attachment — Submitted filename: Response to Reviewers copy.docx [file pone.0350426.s006.docx]

**Response to Reviewers**

We thank the reviewers for their thoughtful and constructive comments, which have significantly improved the quality of this manuscript. We have carefully addressed each concern raised. Below we provide a point-by-point response to all reviewer comments.

**To Reviewer #4:**

Comment: Author mix gender with six... Please unify throughout the full manuscript.
Response: We thank the reviewer for this observation. We have carefully reviewed the manuscript and standardized all terminology to biological sex (Male/Female) throughout the text, tables, and figures to ensure consistency.

Comment: Bad statistical analysis... No regression was done. Needs statistical consultation.
Response: We acknowledge the reviewer's concern regarding the lack of multivariable regression analysis. We agree that logistic regression is the gold standard for identifying independent risk predictors. However, during the revision process, we consulted with a biostatistician and attempted to fit binary logistic regression models for the BAT23 outcome. Due to the fragmentation of the sample across many small subgroups (e.g., specific rare specialties and provinces with n<10) and the resulting quasi-complete separation in the data, the models failed to converge and produced unstable, unreliable estimates.

Given the cross-sectional, descriptive nature of this study in a conflict setting with a finite sampling frame, we opted to retain the robust non-parametric bivariate analyses. To ensure transparency and address this methodological limitation directly, we have added a detailed statement to the **Limitations section of the Discussion** (Page 18, Lines 522-539) explaining the statistical constraints and calling for larger, pooled studies to perform regression analysis in the future. We hope this clarification satisfies the journal's standards for methodological rigor given the context.
