# Supplementary material for: Burnout among Syrian medical residents: A cross-sectional study using the burnout assessment tool (BAT)
Source: PLoS One. 2026 Jun 15;21(6):e0350426. doi: 10.1371/journal.pone.0350426 (PMC13268169; doi:10.1371/journal.pone.0350426)
Supplement: Peer review history

# Peer review: Decision Letter 0

**DOI:** 10.1371/journal.pone.0350426.r001

**Authors:** Abdelaziz Hendy

18 Feb 2026

PONE-D-25-62647

Burnout Among Syrian Medical Residents: A Cross-Sectional Study Using the Burnout Assessment Tool (BAT)

PLOS One

Dear Dr. Al-Jawad,

Thank you for submitting your manuscript to PLOS ONE. After careful consideration, we feel that it has merit but does not fully meet PLOS ONE’s publication criteria as it currently stands. Therefore, we invite you to submit a revised version of the manuscript that addresses the points raised during the review process.

Please submit your revised manuscript by Apr 04 2026 11:59PM. If you will need more time than this to complete your revisions, please reply to this message or contact the journal office at plosone@plos.org. When you're ready to submit your revision, log on to https://www.editorialmanager.com/pone/ and select the 'Submissions Needing Revision' folder to locate your manuscript file.

Please include the following items when submitting your revised manuscript:

- A letter that responds to each point raised by the academic editor and reviewer(s). You should upload this letter as a separate file labeled 'Response to Reviewers'.
- A marked-up copy of your manuscript that highlights changes made to the original version. You should upload this as a separate file labeled 'Revised Manuscript with Track Changes'.
- An unmarked version of your revised paper without tracked changes. You should upload this as a separate file labeled 'Manuscript'.

If you would like to make changes to your financial disclosure, please include your updated statement in your cover letter. Guidelines for resubmitting your figure files are available below the reviewer comments at the end of this letter.

If applicable, we recommend that you deposit your laboratory protocols in protocols.io to enhance the reproducibility of your results. Protocols.io assigns your protocol its own identifier (DOI) so that it can be cited independently in the future. For instructions see: https://journals.plos.org/plosone/s/submission-guidelines#loc-laboratory-protocols. Additionally, PLOS ONE offers an option for publishing peer-reviewed Lab Protocol articles, which describe protocols hosted on protocols.io. Read more information on sharing protocols at https://plos.org/protocols?utm_medium=editorial-email&utm_source=authorletters&utm_campaign=protocols.

We look forward to receiving your revised manuscript.

Kind regards,

Abdelaziz Hendy, Ph.D

Academic Editor

PLOS One

Journal Requirements:

When submitting your revision, we need you to address these additional requirements.

1. Please ensure that your manuscript meets PLOS ONE's style requirements, including those for file naming. The PLOS ONE style templates can be found at

https://journals.plos.org/plosone/s/file?id=wjVg/PLOSOne_formatting_sample_main_body.pdf and https://journals.plos.org/plosone/s/file?id=ba62/PLOSOne_formatting_sample_title_authors_affiliations.pdf

2. Your ethics statement should only appear in the Methods section of your manuscript. If your ethics statement is written in any section besides the Methods, please move it to the Methods section and delete it from any other section. Please ensure that your ethics statement is included in your manuscript, as the ethics statement entered into the online submission form will not be published alongside your manuscript.

3. Please include captions for your Supporting Information files at the end of your manuscript, and update any in-text citations to match accordingly. Please see our Supporting Information guidelines for more information: http://journals.plos.org/plosone/s/supporting-information.

If the reviewer comments include a recommendation to cite specific previously published works, please review and evaluate these publications to determine whether they are relevant and should be cited. There is no requirement to cite these works unless the editor has indicated otherwise.

[Note: HTML markup is below. Please do not edit.]

Reviewers' comments:

Reviewer's Responses to Questions

**Comments to the Author**

1. Is the manuscript technically sound, and do the data support the conclusions?

The manuscript must describe a technically sound piece of scientific research with data that supports the conclusions. Experiments must have been conducted rigorously, with appropriate controls, replication, and sample sizes. The conclusions must be drawn appropriately based on the data presented.

Reviewer #1: Partly

Reviewer #2: Yes

Reviewer #3: Yes

**********

2. Has the statistical analysis been performed appropriately and rigorously?

Reviewer #1: Yes

Reviewer #2: Yes

Reviewer #3: Yes

**********

3. Have the authors made all data underlying the findings in their manuscript fully available?

The PLOS Data policy requires authors to make all data underlying the findings described in their manuscript fully available without restriction, with rare exception (please refer to the Data Availability Statement in the manuscript PDF file). The data should be provided as part of the manuscript or its supporting information, or deposited to a public repository. For example, in addition to summary statistics, the data points behind means, medians and variance measures should be available. If there are restrictions on publicly sharing data—e.g. participant privacy or use of data from a third party—those must be specified.

Reviewer #1: Yes

Reviewer #2: Yes

Reviewer #3: Yes

**********

4. Is the manuscript presented in an intelligible fashion and written in standard English?

PLOS ONE does not copyedit accepted manuscripts, so the language in submitted articles must be clear, correct, and unambiguous. Any typographical or grammatical errors should be corrected at revision, so please note any specific errors here.

Reviewer #1: Yes

Reviewer #2: Yes

Reviewer #3: Yes

**********

5. Review Comments to the Author

Please use the space provided to explain your answers to the questions above. You may also include additional comments for the author, including concerns about dual publication, research ethics, or publication ethics. (Please upload your review as an attachment if it exceeds 20,000 characters)

Reviewer #1: Thank you for this interesting and important work. A few comments:

1) The paper seems to want to link burnout to both working conditions and the crisis conditions in Syria in general. While a potential link certainly makes sense to exist, the data within the paper to do so seems lacking. If there are more opportunities to quote literature to link these, or provide data to backup some of these statements , that would be helpful.

2) Along those lines, burnout seems to be about the same compared to other papers on the topic. One might assume it would be worse in Syria given the situation. It may be helpful to better compare and contrast along these lines.

3) What was the response rate for the survey? ~500 completed the survey out of how many who could have?

4) Please clarify the survey was only for residents (physicians in training) as your methods suggest it was for any first year and beyond physician, which I would define as staff, not a resident.

5) For specialty types, "clinics" is one form, in addition to internal and surgeries. What does that mean?

6) For the table, provinces seem listed haphazardly. Should be alphabetical or by prevalence. The table also seems listed twice.

7) Multiregression analysis may be helpful to see if some of the items that appear significant truly are when taken as a total.

8) In the tables, the items that are significant seem to give a p value across the values, but do not tell us if a particular specialty/province/etc is an outlier compared to the others. You may want to look at this more carefully as it may give richer responses. For example, there is no sig difference for marital status, but that looks like its across 4 domains, whereas what if marital status (regardless of having kids) may have impact, as seem in some other studies.

9) Please review the paper itself. Paragraphs are short with odd formatting. Should consider flow of language.

9) Discussion starts talking about the exposure of war, but this does not seem to be assessed within the study itself. Yet, the discussion makes firm conclusions linking war and burnout. I would suggest softer language or need more data.

Reviewer #2: - note sure why there's two IRB approval number 2527, 2408? need to be fixed, need to be consistent

- "data available based on request" (this need to be removed ), all data need to be provided at the time of submission

- try to cut some repetitive numbers in the discussion and results sections .

- in the conclusion section add part of why this study standout , what make this study unique

- some grammar errors

- add one line at least for the limitation for the study in the discussion part

Reviewer #3: This paper has a good quality for writing and but the references list doesn’tcover the relevant literature adequately and in an unbiased manner. The reference list must be improved. The research include a balanced, comprehensive, and critical view of the research area. The article has a high quality of the content and a large interst for the physicians, that works in the field.

**********

6. PLOS authors have the option to publish the peer review history of their article (what does this mean?). If published, this will include your full peer review and any attached files.

If you choose “no”, your identity will remain anonymous but your review may still be made public.

**Do you want your identity to be public for this peer review?**   For information about this choice, including consent withdrawal, please see our Privacy Policy.

Reviewer #1: No

Reviewer #2: **Yes:**  Abdelwahap Elghezewi

Reviewer #3: **Yes:**  Full professor Anamaria Ciubara

**********

[NOTE: If reviewer comments were submitted as an attachment file, they will be attached to this email and accessible via the submission site. Please log into your account, locate the manuscript record, and check for the action link "View Attachments". If this link does not appear, there are no attachment files.]

To ensure your figures meet our technical requirements, please review our figure guidelines: https://journals.plos.org/plosone/s/figures

You may also use PLOS’s free figure tool, NAAS, to help you prepare publication quality figures: https://journals.plos.org/plosone/s/figures#loc-tools-for-figure-preparation.

NAAS will assess whether your figures meet our technical requirements by comparing each figure against our figure specifications.

# Author comment: Author response to Decision Letter 1

**DOI:** 10.1371/journal.pone.0350426.r002

25 Feb 2026

Dear Dr. Hendy and reviewers,

Thank you very much for the constructive feedback on PONE-D-25-62647. We believe the manuscript is substantially stronger after revising the introduction, methods, results, and discussion to address the points raised below. The revised submission now includes the requested response letter, a tracked-changes version, a clean copy, updated supporting information captions, and a clarified data availability statement with the dataset provided directly.

Reviewer 1

Linking burnout to the Syrian crisis context. We expanded the Introduction and Discussion to cite recent literature describing how protracted conflict, sanctions, and health-system collapse elevate burnout risk, and we explicitly frame our findings within that broader context while acknowledging that our study assesses symptoms rather than exposure to war per se.

• Comparison with other settings. The Discussion now contrasts our 50% BAT red-zone prevalence with both regional and international studies (e.g., Shenoi et al., Bahaa et al.) to clarify where our figures align or diverge and why Syria’s systemic stressors may explain any similarities.

• Response rate. The Methods and Results sections now report the full sampling frame (residents invited via the six governorate registries) and the response rate, emphasizing the representativeness of the cohort.

• Clarify that the survey targeted residents. We have revised the Methods to state that only physicians enrolled in residency programs (postgraduate training years 1+) were eligible, and we removed language that could be interpreted as including staff physicians.

• Definition of “clinics.” We now explain that the “clinical” category covers non-surgical subspecialties such as dermatology, psychiatry, and rehabilitation medicine to differentiate it from purely internal medicine or surgical tracks.

• Province table organization. The province breakdown is now presented once, sorted by prevalence (highest to lowest red-zone rates) with each governorate clearly labeled, and the redundant listing has been removed.

• Multivariable analysis. Because several categorical predictors had sparse cells (especially when subdivided by specialty and province), multivariable regression produced unstable estimates, so we instead added stratified comparisons and emphasized the most robust associations (e.g., internal medicine, surgery, pulmonology/ anesthesia/neurology) while clearly describing the limitations of further adjustment.

• Detailing which categories drive significance. The Results now include descriptive text identifying which specialties, residency years, and workplace factors correspond to the elevated p-values (e.g., internal medicine specialty red-zone rate = 56.4%, mid-level residents have the highest proportion of exhaustion red zone, etc.), and we highlight that marital status did not differ meaningfully across domains.

• Manuscript flow and war exposure language. We smoothed paragraph transitions throughout the Results and Discussion and softened statements that previously linked burnout directly to war exposure, replacing them with phrasing such as “amid the protracted crisis” and noting that we infer broader systemic pressures rather than having measured each stressor.

Reviewer 2

• IRB number consistency. The ethics section in Methods now consistently cites Approval No. 2527, and the standalone ethics statement has been moved entirely into Methods, eliminating the duplicate numbering.

• Data availability. The manuscript no longer states “data available upon request”; instead, All data generated or analysed during this study are included in this published article [and its supplementary information files].

• Reduce repetitive numbers. We trimmed redundant numeric reporting in the Results and Discussion, focusing instead on the most informative comparisons (e.g., we report the median number of shifts once with the associated p-value rather than repeating it across sections).

• Highlight what makes the study unique. The Conclusion now states that this is the first BAT-based nationwide assessment of Syrian residents (across six governorates) that highlights mid-level trainees and high-intensity specialties, underscoring the novel contribution.

• Grammar and language. We proofread the manuscript and corrected grammar issues throughout, particularly in the Discussion and Conclusion.

• Limitations. A limitations paragraph now appears near the end of the Discussion, acknowledging the cross-sectional design, self-reported measures, and the challenges of modeling complex work-environment relationships.

Reviewer 3

• Reference list balance. We expanded the literature cited to include additional recent work on burnout in conflict-affected and resource-constrained settings (e.g., regional cohort studies and systematic reviews) to achieve a more comprehensive and unbiased reference list.

• Critical view of the research area. The Discussion now reflects this broader perspective by acknowledging both the strengths of BAT and the ongoing debates about measuring burnout, thereby aligning with the reviewer’s request for a balanced appraisal.

We hope these revisions satisfactorily address the reviewers’ concerns. The revised manuscript, tracked changes, and supporting documents have been uploaded in accordance with PLOS ONE’s submission requirements.

Attachment Submitted filename: reply to reviewer.docx

# Peer review: Decision Letter 1

**DOI:** 10.1371/journal.pone.0350426.r003

**Authors:** Abdelaziz Hendy

27 Mar 2026

PONE-D-25-62647R1

Burnout Among Syrian Medical Residents: A Cross-Sectional Study Using the Burnout Assessment Tool (BAT)

PLOS One

Dear Dr. Al-Jawad,

Thank you for submitting your manuscript to PLOS ONE. After careful consideration, we feel that it has merit but does not fully meet PLOS ONE’s publication criteria as it currently stands. Therefore, we invite you to submit a revised version of the manuscript that addresses the points raised during the review process.

Please submit your revised manuscript by May 11 2026 11:59PM. If you will need more time than this to complete your revisions, please reply to this message or contact the journal office at plosone@plos.org. When you're ready to submit your revision, log on to https://www.editorialmanager.com/pone/ and select the 'Submissions Needing Revision' folder to locate your manuscript file.

Please include the following items when submitting your revised manuscript:

- A letter that responds to each point raised by the academic editor and reviewer(s). You should upload this letter as a separate file labeled 'Response to Reviewers'.
- A marked-up copy of your manuscript that highlights changes made to the original version. You should upload this as a separate file labeled 'Revised Manuscript with Track Changes'.
- An unmarked version of your revised paper without tracked changes. You should upload this as a separate file labeled 'Manuscript'.

If you would like to make changes to your financial disclosure, please include your updated statement in your cover letter. Guidelines for resubmitting your figure files are available below the reviewer comments at the end of this letter.

If applicable, we recommend that you deposit your laboratory protocols in protocols.io to enhance the reproducibility of your results. Protocols.io assigns your protocol its own identifier (DOI) so that it can be cited independently in the future. For instructions see: https://journals.plos.org/plosone/s/submission-guidelines#loc-laboratory-protocols. Additionally, PLOS ONE offers an option for publishing peer-reviewed Lab Protocol articles, which describe protocols hosted on protocols.io. Read more information on sharing protocols at https://plos.org/protocols?utm_medium=editorial-email&utm_source=authorletters&utm_campaign=protocols.

As the corresponding author, your ORCID iD is verified in the submission system and will appear in the published article. PLOS supports the use of ORCID, and we encourage all coauthors to register for an ORCID iD and use it as well. Please encourage your coauthors to verify their ORCID iD within the submission system before final acceptance, as unverified ORCID iDs will not appear in the published article. *Only*   the individual author can complete the verification step; PLOS staff *cannot*   verify ORCID iDs on behalf of authors.

We look forward to receiving your revised manuscript.

Kind regards,

Abdelaziz Hendy, Ph.D

Academic Editor

PLOS One

Journal Requirements:

If the reviewer comments include a recommendation to cite specific previously published works, please review and evaluate these publications to determine whether they are relevant and should be cited. There is no requirement to cite these works unless the editor has indicated otherwise.

[Note: HTML markup is below. Please do not edit.]

Reviewers' comments:

Reviewer's Responses to Questions

**Comments to the Author**

1. If the authors have adequately addressed your comments raised in a previous round of review and you feel that this manuscript is now acceptable for publication, you may indicate that here to bypass the “Comments to the Author” section, enter your conflict of interest statement in the “Confidential to Editor” section, and submit your "Accept" recommendation.

Reviewer #1: (No Response)

Reviewer #2: All comments have been addressed

Reviewer #3: All comments have been addressed

**********

2. Is the manuscript technically sound, and do the data support the conclusions?

The manuscript must describe a technically sound piece of scientific research with data that supports the conclusions. Experiments must have been conducted rigorously, with appropriate controls, replication, and sample sizes. The conclusions must be drawn appropriately based on the data presented.

Reviewer #1: Yes

Reviewer #2: Partly

Reviewer #3: Yes

**********

3. Has the statistical analysis been performed appropriately and rigorously?

Reviewer #1: Yes

Reviewer #2: I Don't Know

Reviewer #3: Yes

**********

4. Have the authors made all data underlying the findings in their manuscript fully available?

The PLOS Data policy requires authors to make all data underlying the findings described in their manuscript fully available without restriction, with rare exception (please refer to the Data Availability Statement in the manuscript PDF file). The data should be provided as part of the manuscript or its supporting information, or deposited to a public repository. For example, in addition to summary statistics, the data points behind means, medians and variance measures should be available. If there are restrictions on publicly sharing data—e.g. participant privacy or use of data from a third party—those must be specified.

Reviewer #1: Yes

Reviewer #2: Yes

Reviewer #3: Yes

**********

5. Is the manuscript presented in an intelligible fashion and written in standard English?

PLOS ONE does not copyedit accepted manuscripts, so the language in submitted articles must be clear, correct, and unambiguous. Any typographical or grammatical errors should be corrected at revision, so please note any specific errors here.

Reviewer #1: No

Reviewer #2: Yes

Reviewer #3: Yes

**********

6. Review Comments to the Author

Please use the space provided to explain your answers to the questions above. You may also include additional comments for the author, including concerns about dual publication, research ethics, or publication ethics. (Please upload your review as an attachment if it exceeds 20,000 characters)

Reviewer #1: Thank you for the paper and addressing the comments. A few more minor suggestions:

1) Table 1: The specialty order remains unclear. Why is it ordered like that? Would it make more sense to list the specialty types and then under each type, list the specialties attributed to that type, and list them either in alphabetical order or order of frequency? Also, Table 1 has repeated all the data twice.

2) The discussion still has comments that sound like direct causation. Please review in detail as that language likely needs to be softened or backed up with references in the literature.

3) The paper states burnout is the same as a study from the US but the underlying metrics are different. I think this needs to be expanded on as it's a key point (if the underlying metrics are different and you can share that).

4) The discussion suggests measures to mitigate burnout, but this was not the focus of the paper and there is no clear data you raise to say these things will bring improvement. For instance, you first state there is no association with leave days and burnout but then suggest more leave days. While I recognize you discuss why leave days don't help burnout, that does not mean more leave days will resolve the issue.

5) Please review grammar and language use as the discussion remains challenging to follow at times.

Reviewer #2: - The Methods section cites IRB Approval No. 2527, while the Ethics section cites No. 2408

- survey distribution method is not described, no response rate is reported, and the total number of eligible residents contacted is never stated. Without this, the sample's representativeness cannot be assessed

- Type of recruitment rows in Table 3 appear to have the Gov/Private labels transposed, contradicting the baseline data.

- Specialties with n<5 (e.g., Thoracic Surgery n=1, Plastic Surgery n=1) are included in inferential testing. These should be collapsed into "other" or excluded from statistical comparisons.

- All mechanistic claims must be reframed using associative language throughout.

Reviewer #3: This paper has a good quality for writing and but the references list doesn’tcover the relevant literature adequately and in an unbiased manner. The reference list must be improved. The research include a balanced, comprehensive, and critical view of the research area. The article has a high quality of the content and a large interst for the physicians, that works in the field.

**********

7. PLOS authors have the option to publish the peer review history of their article (what does this mean?). If published, this will include your full peer review and any attached files.

If you choose “no”, your identity will remain anonymous but your review may still be made public.

**Do you want your identity to be public for this peer review?**   For information about this choice, including consent withdrawal, please see our Privacy Policy.

Reviewer #1: No

Reviewer #2: No

Reviewer #3: **Yes:**  Full professor Anamaria Ciubara

**********

[NOTE: If reviewer comments were submitted as an attachment file, they will be attached to this email and accessible via the submission site. Please log into your account, locate the manuscript record, and check for the action link "View Attachments". If this link does not appear, there are no attachment files.]

To ensure your figures meet our technical requirements, please review our figure guidelines: https://journals.plos.org/plosone/s/figures

You may also use PLOS’s free figure tool, NAAS, to help you prepare publication quality figures: https://journals.plos.org/plosone/s/figures#loc-tools-for-figure-preparation.

NAAS will assess whether your figures meet our technical requirements by comparing each figure against our figure specifications.

# Author comment: Author response to Decision Letter 2

**DOI:** 10.1371/journal.pone.0350426.r004

31 Mar 2026

Response to Reviewers

We thank the reviewers for their thoughtful and constructive comments, which have significantly improved the quality of this manuscript. We have carefully addressed each concern raised. Below we provide a point-by-point response to all reviewer comments.

Response to Reviewer #1

Comment Response

1. Table 1: The specialty order remains unclear. Why is it ordered like that? Would it make more sense to list the specialty types and then under each type, list the specialties attributed to that type, and list them either in alphabetical order or order of frequency? Also, Table 1 has repeated all the data twice. We thank the reviewer for this observation. The duplicate Table 1 has been removed. Additionally, we have reorganized the specialty list in Table 1 into logical groups (Internals, Surgeries, Clinics, Other) with specialties listed alphabetically within each group for clarity and ease of reference.

2. The discussion still has comments that sound like direct causation. Please review in detail as that language likely needs to be softened or backed up with references in the literature. We have thoroughly revised the Discussion section to replace causal language with associative language throughout. Key changes include: "is attributable to" → "was observed in the context of"; "exacerbated" → "was associated with"; "due to" → "in association with"; "fuels" → "has been linked to"; "highlights the urgent need" → "suggests that further research is warranted." All mechanistic claims have been reframed to reflect the cross-sectional design of the study.

3. The paper states burnout is the same as a study from the US but the underlying metrics are different. I think this needs to be expanded on as it's a key point. We have expanded this section substantially. We now explicitly compare the Burnout Assessment Tool (BAT) used in our study with the Maslach Burnout Inventory (MBI) used by Shenoi et al., highlighting differences in conceptual frameworks (4 dimensions vs. 3 subscales), scoring methods (unified composite score vs. high in ≥1 subscale), and severe burnout rates (50% in our study vs. 21% in Shenoi et al.). We emphasize that direct cross-study comparisons are methodologically challenging and that the numerical similarity in prevalence obscures profound differences in context and drivers.

4. The discussion suggests measures to mitigate burnout, but this was not the focus of the paper and there is no clear data you raise to say these things will bring improvement. For instance, you first state there is no association with leave days and burnout but then suggest more leave days. We have revised the recommendations section to address this concern. The measures are now framed as "potential strategies that merit exploration" rather than definitive solutions. We explicitly note that leave days alone were not significantly associated with burnout severity in this study (rho = -0.057, p = 0.181) and that future interventional studies are needed to evaluate efficacy. The section now emphasizes that these are areas warranting consideration in future research, not conclusions derived from our data.

5. Please review grammar and language use as the discussion remains challenging to follow at times. We have conducted a comprehensive grammar and language review. Overly long sentences have been broken into shorter, clearer statements; transitions have been added where missing; run-on sentences have been split; and redundant phrasing has been removed. The Discussion section now reads more smoothly.

Response to Reviewer #2

Comment Response

1. The Methods section cites IRB Approval No. 2527, while the Ethics section cites No. 2408. We thank the reviewer for identifying this discrepancy. We have verified that the correct approval number is 2527. The Ethics section has been revised to reflect this number consistently throughout the manuscript.

2. Survey distribution method is not described, no response rate is reported, and the total number of eligible residents contacted is never stated. Without this, the sample's representativeness cannot be assessed. We have substantially expanded the Methods section to address this concern. We now clearly describe: (1) the sampling frame (620 eligible residents from master lists obtained from program coordinators); (2) the multi-channel distribution method (institutional email, WhatsApp groups, direct follow-up); (3) the reminder schedule (days 3, 7, and 10); and (4) the response rate (550/620 = 88.7%). The high response rate supports the representativeness of the sample.

3. Type of recruitment rows in Table 3 appear to have the Gov/Private labels transposed, contradicting the baseline data. We thank the reviewer for this careful observation. The labels have been corrected in Table 3. The Governmental row now correctly shows n=412 (76.9%) and the Private row shows n=10 (71.4%), consistent with baseline data in Table 1.

4. Specialties with n<5 (e.g., Thoracic Surgery n=1, Plastic Surgery n=1) are included in inferential testing. These should be collapsed into "other" or excluded from statistical comparisons. We have addressed this by collapsing specialties with n<5 (Hematology, Thoracic Surgery, Plastic Surgery, Laboratory Medicine) into an "Other (collapsed)" category in Table 3. The statistical comparisons (chi-square with Monte Carlo simulation) now respect the assumptions of the test, and the p-value (<0.0001) remains valid.

5. All mechanistic claims must be reframed using associative language throughout. As noted in response to Reviewer #1, we have comprehensively revised the Discussion section to replace causal language with associative language. All claims now reflect associations rather than causal relationships, consistent with the cross-sectional study design.

Response to Reviewer #3

Comment Response

This paper has a good quality for writing but the references list doesn't cover the relevant literature adequately and in an unbiased manner. The reference list must be improved. The research should include a balanced, comprehensive, and critical view of the research area. We thank the reviewer for this constructive feedback. We have substantially improved the reference list by: (1) adding the Maslach Burnout Inventory references (Maslach & Jackson, 1986; Maslach et al., 2001) for proper context when comparing with the BAT; (2) including Shenoi et al. (2018) as the US comparison study; (3) adding a Middle East systematic review (Elbarazi et al., 2017) to provide regional context; (4) completing the incomplete citation for Tuna and Baykal (oncology nurses); (5) ensuring all references are formatted consistently with DOIs where available.

The reference list now provides a balanced and comprehensive view of the literature.

We are grateful for the reviewers' time and expertise, which have helped strengthen this manuscript. We hope the revised version is now suitable for publication.

Attachment Submitted filename: Response to Reviewers.docx

# Peer review: Decision Letter 2

**DOI:** 10.1371/journal.pone.0350426.r005

**Authors:** Abdelaziz Hendy

8 Apr 2026

PONE-D-25-62647R2

Burnout Among Syrian Medical Residents: A Cross-Sectional Study Using the Burnout Assessment Tool (BAT)

PLOS One

Dear Dr. Al-Jawad,

Thank you for submitting your manuscript to PLOS ONE. After careful consideration, we feel that it has merit but does not fully meet PLOS ONE’s publication criteria as it currently stands. Therefore, we invite you to submit a revised version of the manuscript that addresses the points raised during the review process.

Please submit your revised manuscript by May 23 2026 11:59PM. If you will need more time than this to complete your revisions, please reply to this message or contact the journal office at plosone@plos.org. When you're ready to submit your revision, log on to https://www.editorialmanager.com/pone/ and select the 'Submissions Needing Revision' folder to locate your manuscript file.

Please include the following items when submitting your revised manuscript:

- A letter that responds to each point raised by the academic editor and reviewer(s). You should upload this letter as a separate file labeled 'Response to Reviewers'.
- A marked-up copy of your manuscript that highlights changes made to the original version. You should upload this as a separate file labeled 'Revised Manuscript with Track Changes'.
- An unmarked version of your revised paper without tracked changes. You should upload this as a separate file labeled 'Manuscript'.

If you would like to make changes to your financial disclosure, please include your updated statement in your cover letter. Guidelines for resubmitting your figure files are available below the reviewer comments at the end of this letter.

If applicable, we recommend that you deposit your laboratory protocols in protocols.io to enhance the reproducibility of your results. Protocols.io assigns your protocol its own identifier (DOI) so that it can be cited independently in the future. For instructions see: https://journals.plos.org/plosone/s/submission-guidelines#loc-laboratory-protocols. Additionally, PLOS ONE offers an option for publishing peer-reviewed Lab Protocol articles, which describe protocols hosted on protocols.io. Read more information on sharing protocols at https://plos.org/protocols?utm_medium=editorial-email&utm_source=authorletters&utm_campaign=protocols.

As the corresponding author, your ORCID iD is verified in the submission system and will appear in the published article. PLOS supports the use of ORCID, and we encourage all coauthors to register for an ORCID iD and use it as well. Please encourage your coauthors to verify their ORCID iD within the submission system before final acceptance, as unverified ORCID iDs will not appear in the published article. *Only*   the individual author can complete the verification step; PLOS staff *cannot*   verify ORCID iDs on behalf of authors.

We look forward to receiving your revised manuscript.

Kind regards,

Abdelaziz Hendy, Ph.D

Academic Editor

PLOS One

Journal Requirements:

If the reviewer comments include a recommendation to cite specific previously published works, please review and evaluate these publications to determine whether they are relevant and should be cited. There is no requirement to cite these works unless the editor has indicated otherwise.

Please review your reference list to ensure that it is complete and correct. If you have cited papers that have been retracted, please include the rationale for doing so in the manuscript text, or remove these references and replace them with relevant current references. Any changes to the reference list should be mentioned in the rebuttal letter that accompanies your revised manuscript. If you need to cite a retracted article, indicate the article’s retracted status in the References list and also include a citation and full reference for the retraction notice.

[Note: HTML markup is below. Please do not edit.]

Reviewers' comments:

Reviewer's Responses to Questions

**Comments to the Author**

1. If the authors have adequately addressed your comments raised in a previous round of review and you feel that this manuscript is now acceptable for publication, you may indicate that here to bypass the “Comments to the Author” section, enter your conflict of interest statement in the “Confidential to Editor” section, and submit your "Accept" recommendation.

Reviewer #1: All comments have been addressed

Reviewer #4: (No Response)

**********

2. Is the manuscript technically sound, and do the data support the conclusions?

The manuscript must describe a technically sound piece of scientific research with data that supports the conclusions. Experiments must have been conducted rigorously, with appropriate controls, replication, and sample sizes. The conclusions must be drawn appropriately based on the data presented.

Reviewer #1: Yes

Reviewer #4: No

**********

3. Has the statistical analysis been performed appropriately and rigorously?

Reviewer #1: Yes

Reviewer #4: No

**********

4. Have the authors made all data underlying the findings in their manuscript fully available?

The PLOS Data policy requires authors to make all data underlying the findings described in their manuscript fully available without restriction, with rare exception (please refer to the Data Availability Statement in the manuscript PDF file). The data should be provided as part of the manuscript or its supporting information, or deposited to a public repository. For example, in addition to summary statistics, the data points behind means, medians and variance measures should be available. If there are restrictions on publicly sharing data—e.g. participant privacy or use of data from a third party—those must be specified.

Reviewer #1: Yes

Reviewer #4: Yes

**********

5. Is the manuscript presented in an intelligible fashion and written in standard English?

PLOS ONE does not copyedit accepted manuscripts, so the language in submitted articles must be clear, correct, and unambiguous. Any typographical or grammatical errors should be corrected at revision, so please note any specific errors here.

Reviewer #1: Yes

Reviewer #4: No

**********

6. Review Comments to the Author

Please use the space provided to explain your answers to the questions above. You may also include additional comments for the author, including concerns about dual publication, research ethics, or publication ethics. (Please upload your review as an attachment if it exceeds 20,000 characters)

Reviewer #1: (No Response)

Reviewer #4: Author mix gender with six. six is male/female while gender is man/women Please unify throughout the full manuscript

Bad statistical analysis. Many fragmented data with small frequencies. No risk measures were calculated. no regression wan done

Needs statistical consultation

**********

7. PLOS authors have the option to publish the peer review history of their article (what does this mean?). If published, this will include your full peer review and any attached files.

If you choose “no”, your identity will remain anonymous but your review may still be made public.

**Do you want your identity to be public for this peer review?**   For information about this choice, including consent withdrawal, please see our Privacy Policy.

Reviewer #1: No

Reviewer #4: No

**********

[NOTE: If reviewer comments were submitted as an attachment file, they will be attached to this email and accessible via the submission site. Please log into your account, locate the manuscript record, and check for the action link "View Attachments". If this link does not appear, there are no attachment files.]

To ensure your figures meet our technical requirements, please review our figure guidelines: https://journals.plos.org/plosone/s/figures

You may also use PLOS’s free figure tool, NAAS, to help you prepare publication quality figures: https://journals.plos.org/plosone/s/figures#loc-tools-for-figure-preparation.

NAAS will assess whether your figures meet our technical requirements by comparing each figure against our figure specifications.

# Author comment: Author response to Decision Letter 3

**DOI:** 10.1371/journal.pone.0350426.r006

17 Apr 2026

Response to Reviewers

We thank the reviewers for their thoughtful and constructive comments, which have significantly improved the quality of this manuscript. We have carefully addressed each concern raised. Below we provide a point-by-point response to all reviewer comments.

To Reviewer #4:

Comment: Author mix gender with six... Please unify throughout the full manuscript.

Response: We thank the reviewer for this observation. We have carefully reviewed the manuscript and standardized all terminology to biological sex (Male/Female) throughout the text, tables, and figures to ensure consistency.

Comment: Bad statistical analysis... No regression was done. Needs statistical consultation.

Response: We acknowledge the reviewer's concern regarding the lack of multivariable regression analysis. We agree that logistic regression is the gold standard for identifying independent risk predictors. However, during the revision process, we consulted with a biostatistician and attempted to fit binary logistic regression models for the BAT23 outcome. Due to the fragmentation of the sample across many small subgroups (e.g., specific rare specialties and provinces with n<10) and the resulting quasi-complete separation in the data, the models failed to converge and produced unstable, unreliable estimates.

Given the cross-sectional, descriptive nature of this study in a conflict setting with a finite sampling frame, we opted to retain the robust non-parametric bivariate analyses. To ensure transparency and address this methodological limitation directly, we have added a detailed statement to the Limitations section of the Discussion (Page 18, Lines 522-539) explaining the statistical constraints and calling for larger, pooled studies to perform regression analysis in the future. We hope this clarification satisfies the journal's standards for methodological rigor given the context.

Attachment Submitted filename: Response to Reviewers copy.docx

# Editor report: Decision Letter 3

**DOI:** 10.1371/journal.pone.0350426.r007

**Authors:** Abdelaziz Hendy

14 May 2026

Burnout Among Syrian Medical Residents: A Cross-Sectional Study Using the Burnout Assessment Tool (BAT)

PONE-D-25-62647R3

Dear Dr. Al-Jawad,

We’re pleased to inform you that your manuscript has been judged scientifically suitable for publication and will be formally accepted for publication once it meets all outstanding technical requirements.

Within one week, you’ll receive an e-mail detailing the required amendments. When these have been addressed, you’ll receive a formal acceptance letter and your manuscript will be scheduled for publication.

An invoice will be generated when your article is formally accepted. Please note, if your institution has a publishing partnership with PLOS and your article meets the relevant criteria, all or part of your publication costs will be covered. Please make sure your user information is up-to-date by logging into Editorial Manager at Editorial Manager® and clicking the ‘Update My Information' link at the top of the page. For questions related to billing, please contact billing support.

If your institution or institutions have a press office, please notify them about your upcoming paper to help maximize its impact. If they’ll be preparing press materials, please inform our press team as soon as possible -- no later than 48 hours after receiving the formal acceptance. Your manuscript will remain under strict press embargo until 2 pm Eastern Time on the date of publication. For more information, please contact onepress@plos.org.

Kind regards,

Abdelaziz Hendy, Ph.D

Academic Editor

PLOS One

Additional Editor Comments (optional):

Reviewers' comments:

# Editor report: Acceptance letter

**DOI:** 10.1371/journal.pone.0350426.r008

**Authors:** Abdelaziz Hendy

PONE-D-25-62647R3

PLOS One

Dear Dr. Al-Jawad,

I'm pleased to inform you that your manuscript has been deemed suitable for publication in PLOS One. Congratulations! Your manuscript is now being handed over to our production team.

At this stage, our production department will prepare your paper for publication. This includes ensuring the following:

* All references, tables, and figures are properly cited

* All relevant supporting information is included in the manuscript submission,

* There are no issues that prevent the paper from being properly typeset

You will receive further instructions from the production team, including instructions on how to review your proof when it is ready. Please keep in mind that we are working through a large volume of accepted articles, so please give us a few days to review your paper and let you know the next and final steps.

Lastly, if your institution or institutions have a press office, please let them know about your upcoming paper now to help maximize its impact. If they'll be preparing press materials, please inform our press team within the next 48 hours. Your manuscript will remain under strict press embargo until 2 pm Eastern Time on the date of publication. For more information, please contact onepress@plos.org.

You will receive an invoice from PLOS for your publication fee after your manuscript has reached the completed accept phase. If you receive an email requesting payment before acceptance or for any other service, this may be a phishing scheme. Learn how to identify phishing emails and protect your accounts at https://explore.plos.org/phishing.

If we can help with anything else, please email us at customercare@plos.org.

Thank you for submitting your work to PLOS ONE and supporting open access.

Kind regards,

PLOS ONE Editorial Office Staff

on behalf of

Assistant Professor Abdelaziz Hendy

Academic Editor

PLOS One
